# A Review on Antidiabetic Properties of Indian Mangrove Plants with Reference to Island Ecosystem

**DOI:** 10.1155/2019/4305148

**Published:** 2019-12-05

**Authors:** V. Sachithanandam, P. Lalitha, A. Parthiban, T. Mageswaran, K. Manmadhan, R. Sridhar

**Affiliations:** Integrated Island Management Unit, Futuristic Research Division, National Centre for Sustainable Coastal Management, Ministry of Environment, Forests & Climate Change, Government of India, Chennai, Tamil Nadu 600025, India

## Abstract

Mangrove ecosystem has many potential species that are traditionally used by the coastal communities for their traditional cure for health ailments as evidenced by their extensive uses to treat hepatic disorders, diabetes, gastrointestinal disorders, anti-inflammation, anticancer, and skin diseases, etc. In recent times, the diabetes mellitus (DM), a serious physiological disorder all over the world, occur due to the relative or complete deficiency of insulin in the body, characterized by an abnormally high blood glucose level. India has a rich traditional knowledge on plant-based drug formulations that are protective and curative for many health ailments. In this context, we aimed to compile the works done on the antidiabetic activities of mangrove species from Indian coastal regions especially on Andaman and Nicobar Islands as well as some recent works reported from other countries. A total of 126 published articles and 31 mangrove species related pieces of information were gathered with reference to antidiabetic properties of mangroves. This review summarizes the chemical structures, molecular formula, molecular weight, and their biological activities with an aspiration that it might be helpful for the future bioprospecting industries who are interested in develop the natural drugs for DM.

## 1. Introduction

**Figure 1:**
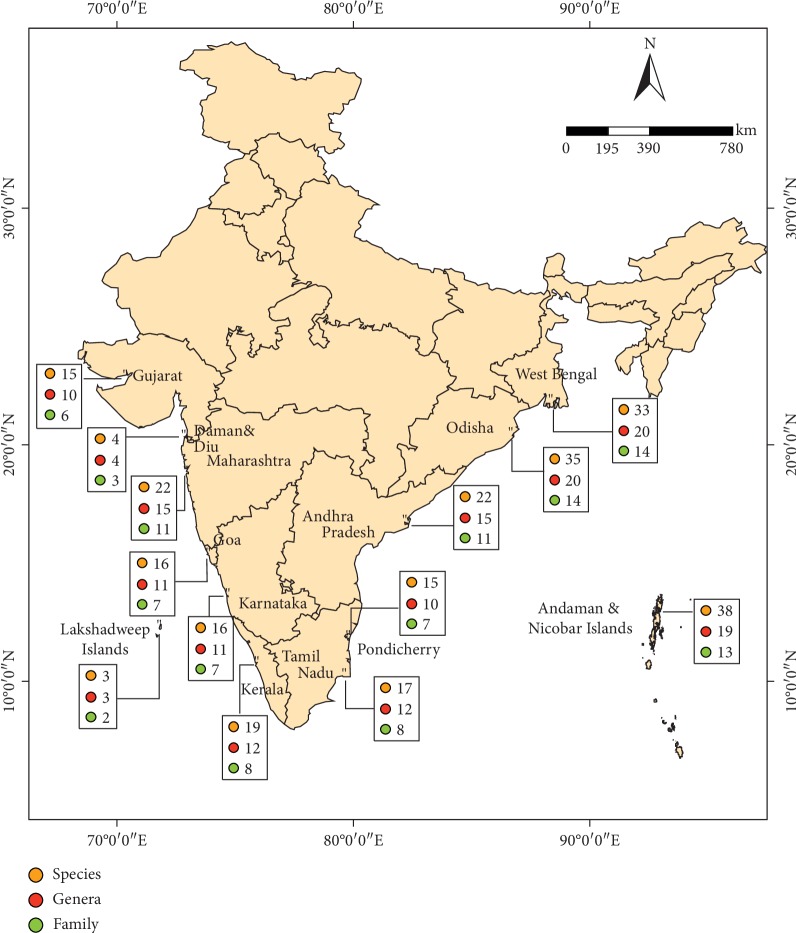


Mangrove species grow at the edge between the coastal and land area in subtropical and tropical regions of the world and are highly adapted to various temperatures, strong coastal winds, extreme tidal waves, salinity fluctuations, coastal water turbulence, river run-off, and anaerobic soil. No other wild species exhibit such physiological and morphological adaptations to the extreme conditions. The worldwide diversity of mangrove flora includes around 81 tree and shrub species of 30 genera from 17 families. Of these, Indian mangroves represent 46 true mangrove species (42 species and 4 natural hybrids) belonging to 14 families and 22 genera [1] (Figure 1). The unique ecology and ecosystem services, plant morphological characteristics, and traditional uses of mangrove plants have already drawn the attention to researchers over the years. Mangroves possess unique biochemical functions in their ecosystem and are considered as a source of novel natural/biological products. Mangroves are rich resources of compounds like polyphenols and tannins. Further, mangrove leaves also possess phenolic compounds, alkaloids, and flavonoids which serve as novel bioactive compounds.

### 1.1. Drugs from Traditional Knowledge

**Figure 2:**
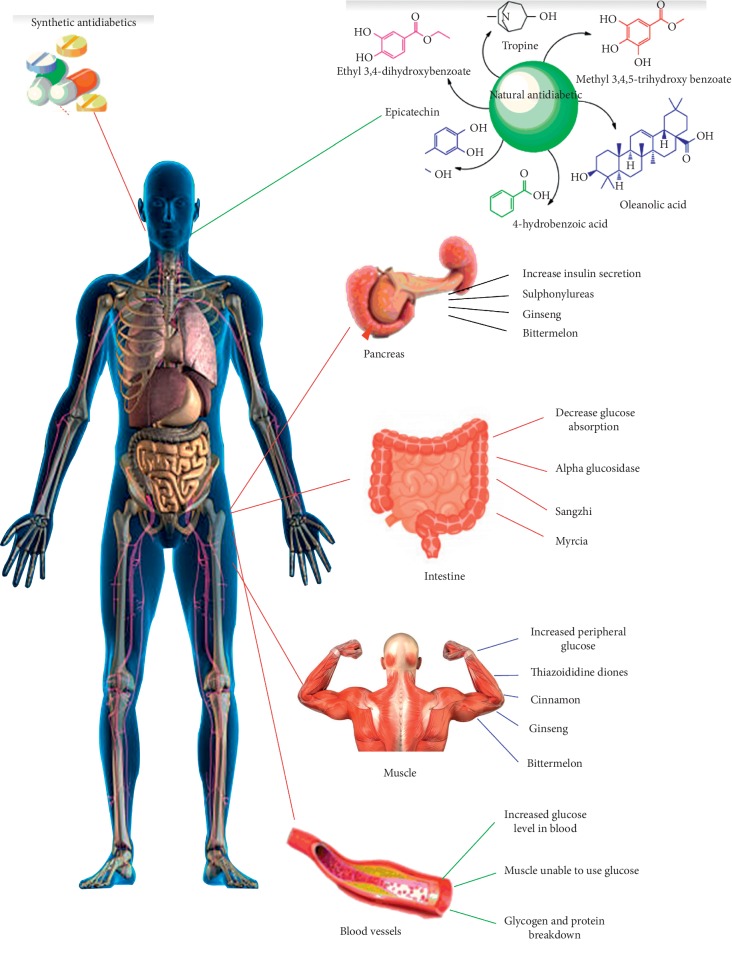


Traditional medicine has long been used as the primary source of therapeutic drugs. The attention on traditional medicine is escalating as there is substantial evidence that it can be a potential source for drugs to combat diseases. The scope and value of traditional medicine research assume greater importance in the realm of healthcare of humankind. In spite of the increased usage of synthetic drugs in modern medicine, half of the world's medicinal compounds are still derived from plants [2]. Some of the most important medicines which have modernized into the modern medical systems have been isolated initially from the plants that were used by ancient society. These wonder drugs include the curare alkaloids, penicillin, and other antibiotics, cortisone [3], reserpine, veratrum alkaloids, podophyllotoxin, and other therapeutic agents [4] (Figure 2). Considering the fact that natural compounds isolated from plants are more economical and may have a holistic effect, assessing traditional knowledge for drug discovery is imperative. Morphine is a well-known product of natural plant medicine.

Drug discovery through traditional knowledge has numerous challenges due to the plethora of ancient and ethnobotanical texts describing myriads of applications of the medicinal plant [5]. Thus, a research program involving potential leads from traditional knowledge and screening based on computational methods like molecular docking, pharmacophore modeling, and molecular dynamics to identify the potential leads and final validation of potential leads through biological studies can be an economical and time-saving approach for drug discovery.

### 1.2. Traditional Knowledge Worldwide: A Brief

Traditional knowledge (TK) based traditional medicines (TM) are generally developed and practiced by primitive communities, based on their medicine experiences, success rate, and depending on numerous trail and errors basis [6]. World Health Organization (WHO) estimates that about 80% of the world population relies on traditional systems of medicine for primary health needs [6]. Scientific literatures from last decade [7–9] and many official factsheets published by WHO stated that, among Asian and African regions, about 85% of the people depend on TM derived by TK practitioners using various primary health care systems [10–12]. Among Southeast Asia, China alone accounts for about 40% depending on TM health care. The recently published data suggested that the use of TK in Asia and African countries is substantially declined due to the lack of documentation of TK since ancient time. The spectrum use of TM has resulted in traditional health use becoming a multinational business between the continents. For instance, in 2012, about 32 billion dollars were spent in the USA on supplementary foods originated from Southeast Asia and Africa, and this may escalate up to 80 billion dollars in 2025.

Every year, medicinal plant-based trade is growing rapidly and India's share in the global market of natural drugs is very low as it contributes only 0.5–1%, whereas demand for these products is increasing at a rapid rate [13, 14]. Developing countries and their traditional people have contributed considerably to the global drugs industry [15].

In India, the epic poems such as Vedas and others illustrated our culture, food, and habitat. The extensive use of TM in the Indian coastal region, composed mainly of plants based derivatives, has been linked to communities' composition and cultural aspects. This is why the WHO and World Bank promotes integrated approaches to documents TK and TM in their healthcare system. In the rural areas of India, 70% of the population is dependent on traditional medicines. Indigenous or traditional knowledge has developed from understanding and documenting the processes in nature. Since ethnobotany is a rapidly expanding science, beginning with the study of plants used by tribals for food, medicine, and shelter, now it includes studies like conservational practices of tribals, ethnopharmacology, ethnopharmacognosy, ethnomusicology, ethnogynaecology, etc. Ethnobotanical studies in relation to traditional communities like the tribal groups have been studied by several researchers in India including the island's ecosystem.

India is rich in its diverse natural resources and one of the seventeen biggest natural biodiversity countries of the world. It has rich vegetation of more than 45,000 plant species, out of which 15,000 to 20,000 plants are estimated to have medicinal values. Out of these, only 7,000 to 7,500 plants are used for the medicinal purpose by established communities [16, 17].

The marine biodiversity is an extremely rich resource for the development of a wide array of goods and services in the food web, pharmaceuticals, cosmetics, coastal protection, etc., but it has been extensively utilized for curing various ailments for many tribal and native communities inhabiting the coastal lines in different parts of the world. As a result of the close and respectful interaction with the marine ecosystem, the indigenous coastal communities possess marine life based rich traditional knowledge that also aids in the sustainable development of the community as well as the marine ecosystem. The valuable traditional knowledge (TK) has so far remained confined within their community and generally passed orally from one generation to another [18]. Various forms of TK, including TM knowledge, have been silently developing over the 19^th^ centuries, with the coastal tribes in nations across the world. Unfortunately, marine TK and TM have been underestimated both commercially and legally. It has still not gained its due importance at the international platform for sustainable use and development of new drugs [18, 19].

### 1.3. Traditional Knowledge of the Andaman and Nicobar Island

Andaman and Nicobar island (ANI), a union territory of the Republic of India, located in the Bay of Bengal, is blessed with the enchanting beauty of whitey sandy beaches, blueish coast, and unique tropical islands with biologically rich flora and fauna [20]. The Andaman and Nicobar archipelago consisting of about 525 islands and islets lies in the Bay of Bengal and forms an arched string stretching geologically from Arakan Yoma in Myanmar in the north to Sumatra in the south (6^o^4′and 13^o^ 41′N latitude and 92^o^11′ and 94^o^10′E longitude) with a land area of 8290 km^2^. Floristically, there are 2654 species belonging to 1083 genera and 237 families, of which 308 species are endemic. The native people of these islands belong to two races, namely, Onges, Jarawas, Sentinelese, and Great Andamanese from Andaman Islands are probably the most primitive communities in India. The Nicobarese and the Shompen tribes are confined to Nicobar group of islands [20]. The forest resource of the islands has a rich repository of biodiversity of medicinal plants representing invaluable ethnobotanical wealth from the six indigenous tribes, namely, the Great Andamanese, Onges, Jarawas, Sentinelese, Nicobarese, and Shompens. Among them, the Great Andamanese, Onges, Jarawas, and Sentinelese originated from the Andaman Islands are probably the most primitive communities in India. The Nicobarese and the Shompen tribes are confined only to Nicobar group of islands [21].

The island's tropical forests represent nature's major storehouse of chemicals and pharmacodynamics compounds used in the perfumery, cosmetics, and pharmaceutical industries. The folklore medicinal uses of the tribes except for the Sentinels have been documented by various researchers [16, 17, 22–26]. Unfortunately, the traditional healing systems and knowledge of these aboriginals have largely eroded along with the natural resources, because of the lack of needed support and recognition, as well as the rapid destructions of their habitats through a series of unsustainable developmental activities such as urbanization, natural calamities, and sea level rise impact on small islands. Further, due to the exposure of these tribes with the outside people, modern lifestyle, education, and lack of time, they gradually forget their TK practices and culture and TM benefits over many decades [20]. Besides all these parameters, the aged TK practitioners who fully depend on the TM are putting their efforts in the transformation of knowledge from one generation to another, but due to the lack of interest in the younger generation, the values of TK and TM practices face the threat of getting vanished. In addition, ANI is located in the seismic hard zone of IV, which is more prone to the earthquake and tsunami waves. The 2004 Tsunami hit ANI very aggressively and 70% of coastal ecosystems were highly damaged, which led to the destruction of coastal habitats and more damages to inland areas.

In these circumstances, to preserve the genetic resources of the medicinal plants for the universal sustainable utilization, the IUCN has proposed a medicinal plant specialist group for making public awareness of ethnobotanical uses and for the conservation of these plant groups in the threating areas. The above statement clearly depicts that there is an urgent need to found out the entire uses of ethnomedicinal plants used by different tribes of the Indian islands. It is also important that such bioresources are conserved and used sustainably, as the island inhabitants still source these plants from the wild for the treatment of ailments. Study of the biogeography of at least some of such medicinal plant species could be useful for further management practices. Although many research works have been carried out in the landward side for medicinal plant resources and its conservation, there is a need for the conservation and sustainable use of medicinal plants of the coastal forest areas. In this review, we attempt to cover research information of the documentation of indigenous knowledge on coastal plants and recent investigations on the biological activities of mangroves extracts especially on the antidiabetic properties, retrieved from different web sciences that focused on drug molecules identification, future perspective, research implication, and conservation of mangroves resources of ANI.

### 1.4. Mangrove Plant-Based Bioactive Studies

Traditionally, mangrove plants are used in folklore medicine for the treatment of several ailments including diabetes throughout worldwide [27]. Many plants are considered to be a rich source of potent antidiabetic drugs, and these herbal preparations are considered to be devoid of any side effects. Approximately, 400 plants and their secondary metabolites, namely, alkaloids, carotenoids, flavonoids, glycosides, polyphenolic, terpenoids, and tannins molecules, were used for treating DM [28, 29].

In the recent era, people from developed and developing countries are increasingly being diagnosed with diabetes. In 2016, WHO reported that approximately 400 million people globally suffer from diabetes disorder that caused about 1.6 million deaths in 2015. Further, the WHO has projected that the diabetes population will likely to be increased to 300 million in 2025 [30]. The current trends in India indicated that there is an alarming rise in the prevalence of diabetes which has gone beyond epidemic form to a pandemic one. Globally, diabetes outbreaks place an enormous amount of public health disorders. The occurrences and consequences associated with DM are found to be in high risk for countries like India (31.7%), China (20.8%), and the USA (17.7%) as reported by Balaraman et al. [6]. From this data, it is projected that by 2030, India, China, and the USA will have the largest number of people with DM [31]. Instead of being a single disorder, DM shows a series of disorders, characterized by increased fasting, postprandial glucose concentration, insulin deficiency or decreased insulin action and impaired glucose tolerance, and malfunction in lipid and protein metabolism. The long-term use of commercially available drugs for the cure of diabetes may also cause unwanted side effects. As a result, many studies are underway to find natural remedies that can effectively reduce the intensity of diabetes [32].

Therefore, the management of DM in recent times possesses a big challenge throughout the world. Not only insulin but also several types of drugs that act to reduce blood glucose (insulin secretagogues, insulin sensitizers, *α*-glucosidase inhibitors, peptide analogues, dipeptidyl peptidase-4 inhibitors, and glucagon-like peptide-1) have been developed by current medicinal scenario. However, these synthetic oral hypoglycemic agents possess characteristic profiles of serious side effects like hypoglycemia, weight gain, gastrointestinal discomfort (disorder), nausea, diarrhea, liver function disorder, jaundice, heart failure, etc. [33]. Therefore, alternative treatment way is the need of the hour.

A set of associated diseases (blood pressure etc.) in which the body cannot regulate the amount of sugar in the blood is called DM. The blood delivers glucose to provide the body with energy (sugar molecules) to perform a person's daily activities. The food a person eats is converted into glucose by the liver, thereby releasing the glucose into the bloodstream. In a healthy person, the blood glucose level is regulated by several hormones, primarily insulin secretion from pancreatic *β*-cell, a small organ between the stomach and liver. It also makes other important enzymes which are released directly into the gut and help digest food. Insulin allows glucose to exit from the blood into cells throughout the body, where it is used for fuel/energy. DM either does not produce enough insulin or cannot use insulin properly or both. In the disorder, blood glucose cannot move efficiently into cells, so blood glucose levels remain high. This not only starves all the cells that need glucose for fuel but also harms certain organs and tissues exposed to the high glucose levels [34]. Categorically, there are two types of DM recognized by the WHO, namely, Type 1 diabetes (insulin-dependent) and Type II diabetes (noninsulin-dependent). Treatment of diabetes is considered as the main global problem and successful treatment is yet to be discovered. Two major drugs like (i) insulin and (ii) oral hypoglycemic agents which are the first line of treatment for diabetes have some side effects and fail to significantly alter the course of diabetic complications [35].

Gurudeeban et al. showed that the crude extracts of *Citrullus colocynthis, Aegle marmelos,* and *Ipomoea pes-caprae* exhibited potential *α*-glucosidase inhibitory activity. These three plants can be exploited to treat diabetes [36]. Similarly, 5 different compounds (cysteine, phenylacetic acid, acrylamide, caprylone, and oleic acid) isolated from *Rhizophora mucronata* were evaluated for an inhibitory action on DPP IV inhibitors using in silico approach [37]. In 2014, methanolic extract of *Rhizophora apiculata* yielded 18 phytocompounds. The results of GC-MS identified 18 phytocompounds, among those major peaks were 1-adamantyl-*p*-methylbenzalimine, clivorine, 4-butyl pyridine, 1-oxide, acetamide, and *p*-aminodiethyl-amidine. These major compounds were subjected to in silico analysis on human peroxisome proliferator-activated receptor gamma protein determined by Auto DOCK 4.0 and identified as thiazolidinediones [38]. Selvaraj et al. reported that *α*-glucosidase is the key intestinal enzyme having clinical relevance in the treatment of DM. In the study, leaf extract of *R. apiculata* contains a huge amount of alkaloids and exhibited significant *α*-glucosidase inhibitory activity (250.53 ± 0.51 mg/g) [39].

Recently, it was reported that alkaloid compound like glycosine is derived from ethanolic extract of *R. apiculata*. It showed antidiabetic/antihyperglycemic effect of glycosine in diabetic rats. The results showed that glycosine treatment significantly (*p* < 0.01) reduced the blood glucose level and increased the body weight and hemoglobin levels, high-density lipoprotein and insulin levels, protein, and the activity of hexokinase when compared to untreated rats. Decreased activities of liver function enzymes as well as the level of urea and creatinine were observed in glycosine treated rats [40]. Selvaraj et al. exhibited dichloromethane fraction (DCM-F) of *R. mucronata* on noninsulin-dependent diabetes mellitus. 100 mg/kg of DCM-F treatment in diabetes rats stimulates the action of *β*-cells to secrete insulin and improve antihyperglycemic conditions in NIDDM. This is also clearly evident from the carbohydrates lipid profile, plasma insulin, and marker enzymes present in the serum [41]. Selvaraj et al. revealed that *Aegiceras corniculatum* leaf extracts showed a moderate reduction in blood glucose (382 ± 34 to 105 ± 35), glycosylated hemoglobin, a decrease in the activities of glucose-6-phosphatase and fructose-1,6-bisphosphatase, and an increase in activity of liver hexokinase active through the oral administration of extract of 100 mg/kg [42]. Satyavani et al. revealed that medicinally important mangrove species such as *Acanthus ilicifolius, Excoecaria agallocha, R. apiculata*, and *R. mucronata* were extracted for secondary metabolites with different solvents such as petroleum ether, diethyl ether and ethanol. A total of 135 chemical constituents were identified and compared with retention time in the NIST library in 2011. The chemical constituents were characterized into essential oils, higher alkanes, acid, alcohol, and esters. Major peaks indicated the presence of 8-pentadecane, 1, 2, 5-trimethylphyrrole, di-(2-ethylhexyl) phthalate, diethyl phthalate, epoxyhexobarbital, and cyclooctacosane [43].

Kaliamurthi et al. [44] reported on 33 medicinal plant species and documented the hypoglycemic and wound healing properties of plant species especially halophytes and its associates collected from the coastal village of Kodiyampalayam from Southeast coast of India. Kaliamurthi and Selvaraj [45] conducted several studies and elaborated the risk factors responsible for Type 2 DM including obesity, hypertension, smoking, physical inactivity, low education, dietary patterns, family history, and specific gene. Recently, researchers focused their interest on finding out the potential antidiabetic molecules from the medicinal plants to reduce the side effects caused by commercial drugs. Antinociceptive effects of *E. agallocha* against chemically and thermally induced nociception was studied on Albino mice which received a dosage of 10, 15, 20, or 25 mg/kg of alkaline chloroform fraction (Alk-CF). Compared with controls, Alk-CF decreased the writhing numbers (*p* < 0.01) in a dose-dependent manner [45]. Similarly, results showed an antinociceptive effect in mice of thillai flavonoid rutin [42].

## 2. Materials and Methods

This review was carried out by collecting information on relevant research findings with the help of Internet search engines like Google, Google Scholar, PubMed, ScienceDirect, and ResearchGate and other published articles, reports, and monographs. A total of 126 published articles have been reviewed and the related information was gathered for this current study with respect to antidiabetic research from Indian coastal region and from other countries.

### 2.1. Antidiabetic Agents from Mangrove Plants

Alkaloids brugine are 2-dithiolane (sulfur-containing) compounds, which have been isolated from *Bruguiera sexangula* (Table 1 (Sl. No. 1-2)). Three sulfur compounds along with an alkaloid brugine were reported from the stem and bark of *B. cylindrica* by Japanese scientists during 1975–1976 [50]. Similarly, the presence of acanthicifoline in *A. ilicifolius* and brugine (a sulfur containing alkaloid) in *B. sexangula* was reported by Katu and Takahashi and Richter et al., respectively [46, 47]. This study showed that *Bruguiera* sp. exhibited high anticancer activity and antidiabetic activities [48, 50].

Loder and Russell [50] identified the presence of alkaloids (tropine 2 and tropine esters of acetic acid, isobutyric acid, isovaleric acid, propionic acid, *n*- butyric acid, benzoic acid, and tropine esters of ethyl 3,4-dihydroxybenzoate) (Table 1 (Sl. No. 3–10)) in the stem and bark extracts of *B. sexangida* [50, 51]. Tropine alkaloids are medicinally useful natural products and their synthetic derivatives show anticancer, antiemetic drugs, antispasmodics, mydriatics, and cholinergic muscarinic antagonists [52].

Bioactive molecules of polysaccharides from *S. alba* are mainly derived from the seeds and have been reported to possess antidiabetic properties [53, 54]. In addition to that, complex polysaccharides show various biomedical applications such as antimicrobial, antiviral, and antihyperglycemic agent and proliferation activity for fibroblasts [55, 56]. Rutin, quercetin, kaempferol, catechin, and (-)-epicatechin in Table 1 (Sl. No. 11–15) represent flavonoids that are abundantly found in a mangrove plant species such as *R. apiculata* and *A. ilicifolius* [49]. *A. marina*, *Xylocarpus granatum,* and *B. sexangula* are reported to be rich in flavonoid compounds, namely, rutin, quercetin, kaempferol, catechin, and epicatechin that exhibited hypoglycemic activities and other biological activities such as antibacterial, antifungal antimycobacterial, antimalarial, antiretroviral, and antiviral activities [59–62].

As can be seen in Table 1 (Sl. No. 16–20), *β*-sitosterol (beta-sitosterol) **16**, *β*-amyrin **17**, *α*-amyrin **18**, ursolic acid **19**, and stigmasterol **20** are several phytosterols (plant sterols compounds) with chemical structures similar to that of cholesterol. These compounds (Figures D **16** and **17**) are derived from *B. gymnorhiza,* and *B. sexangula* [45, 63]. These sterols showed high anti-inflammatory activity, antidiabetic effects, inducing apoptosis, angiogenic effect, hypocholesterolemic activity, antioxidant effects, and anthelminthic and antimutagenic activities as reported by Soodabeh et al. [64].

Sun and Guo [69] first documented the presence of bartogenic acid **21** from the extract of stem, bark, and fruits of *Barringtonia racemosa* ethanolic extract. Further studies have verified the presence of bartogenic acid by K. R. Patil and C. R. Patil [70]. Bartogenic acid shows anti-DM, antiarthritic activity, antitumor, antinociceptive, antibacterial, and antifungal activities and anti-inflammatory drugs [69, 71].

Hexane and chloroform extracts of the leaves of *Scyphiphora hydrophylacea* yielded compound, namely, oleanolic acid **22**. Oleanolic acid has been isolated for the first time in Sri Lanka and demonstrated in vitro cytotoxic effects in estrogen receptor-positive (MCF-7) and nonsmall lung cancer (NCI-H-292) cells [72]. Triterpenoid has been reported to show anti-inflammatory, antitumor, hepatoprotective, antidiabetic, and antibacterial properties [72–74].

Methyl 3,4,5-trihydroxy benzoate **23**, gallic acid **24**, and 3,4,5-trimethoxy phenyl 1-OF (6- galloyl)-glucopyranoside **25** are phenolic compounds obtained from *B. racemosa, Rhizophora* sp., and their biological activities such as antidiabetic, antibacterial, antifungal, antiviral, anti-inflammatory, antioxidant, and anticancer activities [65–68, 74]. This review highlights the traditional knowledge of mangroves plants that possess numerous bioactive compounds which are summarized in Table 1.

### 2.2. Studies on Antidiabetic Activities of Mangroves Plants

#### 2.2.1. Antidiabetic Activity of Mangrove Extracts and Their Phytochemicals

Mangrove plants are considered to be a rich source of potent antidiabetic agents and are considered to be devoid of side effects. It is estimated that more than 400 plants and their secondary metabolites such as glycosides, alkaloids, terpenoids, flavonoids, carotenoids, tannins, polyphenolic, aliphatic alcohols, acids, amino acids, carbohydrates, hydrocarbons (including polyunsaturated fatty acids), lipids, pheromones, phorbol esters, and steroid derivatives are being used for the management of diabetes mellitus across the globe [27, 28]. Mangroves are woody plants growing at the interface between the land and sea in tropical and subtropical latitudes, where they exist under conditions of high salinity, extreme tides, strong winds, high temperatures, and muddy, anaerobic soils. Asia is the richest region of mangrove species diversity with 44 species reported [49]. Some of the important mangrove plants along with their phytochemical constituents and their mechanism of antidiabetic achievement are shown in Table 2.

#### 2.2.2. Acanthaceae

Acanthaceae is represented by two genera, namely, *Acanthus* and *Avicennia*. The genus *Acanthus* is represented by three species in India, namely, *A. ilicifolius*, *A. ebracteatus,* and *A. volubilis*. Of these, *A. ilicifolius* is commonly distributed, while *A. ebracteatus* is found only in Kerala, Puducherry, and ANI at confined locations [1]. Venkataiah et al. reported that ethanolic root extract of *A. ilicifolius* at a dose of 200 and 400 mg/kg body weight significantly reduced the blood sugar level in normal glucose-fed hyperglycemic and alloxan-induced diabetic rats. Regeneration of *β*-cells has also been reported in diabetic rats [78]. Flavonoids, alkaloids, terpenoids, tannins, and steroids are present in the root extracts of this plant and this may play an important role in their hypoglycemic activities [56].

#### 2.2.3. Arecaceae

Arecaceae family is represented by two species, namely, *Nypa fruticans* and *Phoenix paludosa*. In India, both are distributed in Sundarbans and ANI. TK of *N. fruticans* and its uses for different ailments by the local practitioners/coastal communities of southern regions of Bangladesh and ANI has been well documented [16]. This species has very limited distribution in the Indian coast. However, the methanolic leaf and stem extracts of *N. fruticans* (500 mg/kg) have been reported to show their significant antihyperglycemic effect in diabetic mice [114].

#### 2.2.4. Avicenniaceae

The family Avicenniaceae is represented with genus *Avicennia* by three species, namely, *A. alba*, *A. marina*, and *A. officinalis,* and all are commonly populated in the mangrove habitats of India. Recent studies reveal that the ethanolic extract of *A. marina* leaf has antihyperglycemic activity in alloxan-induced diabetic rats. In addition, when the ethanolic leaf extract (250 and 500 mg/kg) was used for the treatment of the diabetic rats for 15 days, it resulted in a significant reduction in the blood glucose levels along with an increase in total hemoglobin (Hb), total protein, and serum insulin. The leaf extract can reduce the level of serum urea that confirms the capacity to protect vital tissues, kidney, liver, and pancreas. In addition, it also improved the biochemical parameters such as serum phosphorus, albumin, and globulin. The possible mechanism underlying the antihyperglycemic action of *A. marina* is attributed to the stimulation of surviving *β*-cells that is releasing more insulin [118]. Mahera et al. [119] reported that methanolic extract of pneumatophores (aerial roots) of *A. marina* exhibits antihyperglycemic effect, which might be due to the inhibition of AGEs. Aljaghthmi et al. [79] elucidated the antidiabetic properties present in *A. marina*.

#### 2.2.5. Euphorbiaceae

Two species, namely, *E. agallocha* and *E. indica,* belonging to the genus *Excoecaria* representing Euphorbiaceae are recorded in Indian mangroves. *E. agallocha* is commonly distributed in back mangrove areas where there is lower salinity, whereas *E. indica* is found in Sundarbans, Odisha, and Kerala. Recently Ragavan et al. reported *E. indica* species from mangrove ecosystem of ANI [1].

The methanolic stem extract of this plant has shown to reduce serum glucose levels in doses of 200 to 400 mg/kg [88]. This activity was significantly lower compared with glibenclamide (10 mg/kg·bw), an antidiabetic drug closely related to sulfonamide antibiotics. *E. agallocha* has also been found to contain *b*-amyrin acetate which is thought to be responsible for its antidiabetic activity [120]. Thirumurugan et al. [89] revealed that ethanolic leaf extracts of *E. agallocha* species possessed significant hypoglycemic activity. Flavonoids, triterpenoids, and phenolic compounds are the bioactive principles responsible for antibiotic and antidiabetic potential compounds which are present in *E. agallocha* species.

#### 2.2.6. Malvaceae

Malvaceae is represented by two genera, namely, *Brownlowia* and *Heritiera* in Indian mangroves. Two species of genus *Heritiera,* namely, *H. fomes* and *H. littoralis,* are known from Indian mangroves, of which the former is reported only from Sundarbans and Odisha, but due to the reduction in freshwater input in both places, it has become rare. *H. littoralis* is known from Odisha, Maharashtra, and ANI. Dose-dependent reductions in the concentration of serum glucose in mice upon treatment with crude methanol extract of *H. fomes* bark are utilized for antidiabetics. At 60 minutes following glucose administration, *H. fomes* bark extract (250 mg/kg) significantly lowered blood glucose levels by 49.2% compared to 43.5% by glibenclamide (antidiabetic drug). It was further reported that the crude methanolic extract possesses long-term action in its glucose-lowering effect and is also better than glibenclamide drug [91]. In the future, there is an in-depth study to be needed on phytochemicals speciation of *H. littoralis* from Indian mangroves. In ANI, *H. fomes* and *H. littoralis* are common in both groups of islands [1]. There is a need for more in-depth study on the bioactive compounds from the two species of genus *Heritiera* from ANI as well as Indian mangrove.

#### 2.2.7. Rhizophoraceae

The family Rhizophoraceae constitutes the four genera *Bruguiera, Ceriops, Kandelia* and *Rhizophora* representing Rhizophoraceae that are found in Indian mangroves.

#### 2.2.8. Bruguiera

There are six species present in this genus as reported throughout the world. Four species of genus *Bruguiera,* namely, *B. gymnorrhiza, B. cylindrica, B. parviflora,* and *B. sexangula,* are reported from India. Of these, the first two species are commonly distributed in Indian mangroves, while *B. parviflora* is restricted to Sundarbans, Odisha, ANI, and Maharashtra. Bark part of *B. gymnorrhiza* was extracted with ethanol solvent which displayed antihyperglycemic effect in streptozotocin- (STZ-) induced diabetic rats. Treatment with ethanolic bark extracts (400 mg/kg) for 21 days reported significant reduction in blood glucose level in the STZ-induced diabetic rats, which was comparable to that of standard drug glibenclamide (0.5 mg/kg). Further, a decreased level of total cholesterol, triglycerides, very-low-density lipoprotein, and low-density lipoprotein along with increased high-density lipoprotein level in the diabetic rats was observed [121]. Oral administration of ethanolic extract of *B. gymnorrhiza* normalized the levels of blood glucose in rats. The potent antidiabetic effect of the plant extract suggests the presence of various potent antidiabetic active compounds, which produced an antihyperglycemic effect in diabetic rats. The present literature survey observed that different compounds such as bruguierol, *β*-sitosterol, *α*-amyrin, *β*-amyrin, lupeol, oleanolic acid, ursolic acid, taraxerol, gymnorhizol, and ellagic acid were isolated from *B. gymnorrhiza* plant and its potent antidiabetic activity.

#### 2.2.9. *Ceriops* sp

Rhizophoraceae includes the genus *Ceriops* that constitutes two species; *C. decandra* and *C. tagal* are common in Indian mangroves. Both species have been reported from ANI. A significant serum glucose level lowering capacity was marked in alloxan-induced diabetic mice (animal experiment model) upon oral administration of ethanolic leaf extract taken from *C. decandra* (120 mg/kg) developed by Nabeel et al. [32]. This capacity was comparable to that of glibenclamide (0.1 mg/kg bw). The increase in insulin secretion, body weight, and hemoglobin (Hb A1c) levels and decrease in HbA1c levels in diabetes-induced rats were due to ethanolic leaf extract of *C. decandra* treatments. The ethanolic *C. decandra* leaf extracts were found to be involved in the regulation of hexokinase, glucose-6-phosphatase, and fructose-1,6-bisphosphatase which play a key role in glucose metabolism in mitochondria organelles. Normal levels of glucose-6-phosphatase and fructose-1,6-bisphosphatase activities were observed in diabetic rats upon the administration of ethanolic *C. decandra* leaf extracts. Further, diabetic rats also showed increased hexokinase activity. Antihyperglycemic action of *C. decandra* can be attributed to its ability in stimulating surviving *β*-cells in pancreases to produce more insulin [32]. A study by Tiwari et al. concluded that treatment with ethanolic leaf extracts of *C. tagal* improved glucose tolerance of normoglycemic rats after sucrose load. The treatment also lowered blood glucose levels upon a 250 mg/kg oral dose in STZ-induced diabetic rats. The application of hexane subfraction of ethanolic extract of *C. tagal* (100 mg/kg) in normal healthy rats after sucrose load proved to be most effective for antiglycemic activity. This can be compared to the effect of metformin. Unique compounds isolated from *C. tagal* hexane fraction showed significant inhibition against protein tyrosine phosphatases (PTPase) enzyme activity which is involved in insulin action in Type 2 diabetes [85]. Tamrakar et al. [86] reported that n-hexane soluble fraction of ethanolic leaf extracts of *C. tagal* stimulates glucose uptake in L6 muscle cells in a dose-dependent manner, which is comparable to metformin standard. Similarly, Lawag et al. revealed that antihyperglycemic activity of hydroalcoholic bark of *C. tagal* is due to its *α*-glucosidase inhibition potential [87].

#### 2.2.10. *Rhizophora* sp

Three out of the ten species of *Rhizophora* genus (*R. apiculata, R. annamalayana,* and *R. mucronata*) possess antidiabetic activity [56]. At 250 mg/kg doses, the ethanolic root extracts of *R. apiculata* showed promising antihyperglycemic activity in experimental rats. A large number of phytochemicals are found in the chloroform and aqueous subfractions of ethanolic root extract accounting for its antihyperglycemic activity. After purification, seven compounds were isolated: lupeol, oleanolic acid, *β*-sitosterol, palmitic acid, *β*-sitosterol-*β*-D-glucoside, inositol, and pinitol. Among these, inositol and pinitol showed promising activity in the STZ model with 100 mg/kg dose level [93]. The ethanolic leaf extracts of *R. apiculata* showed hypoglycemic and antihyperglycemic activities in normal, glucose-fed, and STZ diabetic rats [94]. The hypoglycemic action *of R. apiculata* is due to the presence of flavonoids along with other bioactive compounds. The antidiabetic properties of the hydromethanolic leaf extracts of *R. apiculate* are due to its radical scavenging and *β*-cell protection properties. Nabeel et al. have reported the antidiabetic potential of *R. mucronata, R. apiculata, and R. annamalayana* [56, 92]. 60 mg/kg *Rhizophora* aqueous extract was orally administered in alloxan-induced diabetic rats. The results revealed that it aided in modulation/acceptable normal levels of blood glucose level. The noticeable antidiabetic activity was observed from the extract of *R. apiculata* in comparison to other mangrove extracts [92]. SDS-PAGE analysis elucidated the presence of insulin-like protein in the mangrove extracts and confirmed that assertion was done by an enzyme-linked immunosorbent assay (ELISA) [56]. Therefore, the antidiabetic property in *R. apiculate* is clearly noted due to the secretion of insulin-like protein biomolecules and its action against reducing the blood glucose level [92].

Gaffar et al. reported the antidiabetic activity of *R. mucronata* and revealed that it is a plant's sole capacity to inhibit carbohydrate digestion and absorption of glucose biomolecules [95]. Haque et al. [96] reported that the bark extracts of *R. mucronata* aqueous layer have hypoglycemic and antihyperglycemic activities. The bark extracts showed dose-dependent antidiabetic effects which helped suppress postprandial hyperglycemia. The most probable mechanism behind the hyperglycemic effect is glucose absorption inhibition. A similar study by Lawag et al. revealed that antihyperglycemic activity of hydroalcoholic bark of *R. mucronata* is due to its *α*-glucosidase inhibition potential observed. Traditionally, *R. mucronata* was utilized to cure diabetes [87].

#### 2.2.11. *Kandelia candel*

The genus *Kandelia* is represented by two species, namely, *K. candel* and *K. obovata* of the family Rhizophoraceae in mangrove communities, of which the former is known from both east and west coasts of India and ANI. Mangrove species *Kandelia* mostly occurs in a middle zone of the mangrove ecosystem. Bark and leaves are used in the treatment of DM in different coastal regions of India. The bark of *K. candel* is suitable for an industrial application like tanning heavy leather and for dyeing in red and brown colours production. Further, phytochemical analysis of the *K. candel* species shows flavonoid, glycosides, triterpenoids, tannins, saponins, and polyphenols compounds. However, there is no availability of comprehensive study on purified compounds of *K. candel* from Indian coast [97].

#### 2.2.12. Lythraceae

Two genera, namely, *Pemphis* and *Sonneratia,* representing Lythraceae are found in Indian mangroves. In India, the genus *Sonneratia* is represented by seven species, namely, *S. alba, S. caseolaris, S. griffithii, S. ovata, S. lanceolata, S. urama,* and *S. gulngai*. Of these, *S. ovata, S. lanceolata, S. urama,* and *S. gulngai* are new records for India from the ANI. Out of the seven identified species of genus *Sonneratia*, antidiabetic activity has been reported in three species, namely, *S. alba*, *S. apetala,* and *S. caseolaris*. Morada et al. reported the antidiabetic potential of methanolic leaf extracts of *S. alba* using *in vivo* mice model. The extreme blood-glucose-attenuating activity of the extract was related to complex polysaccharide molecule obtained from *S. alba* leaf extracts. Significant reduction in sugar level was observed during the first 6 (19.2%) and 12 h (66.9%) after the administration of the extracts to the diabetic mice [55]. Fruits of *S. caseolaris* have many therapeutic applications in folklore medicine [61]. Compounds such as oleanolic acid, *β*-sitosterol-*β*-D-glucopyranoside, and luteolin, which are isolated from the methanolic extract of its fruits, have shown inhibition of the *α*-glucosidase enzyme in a dose-dependent manner [98]. Further, the methanolic fruit extracts of this plant significantly reduced the serum glucose concentrations in mice loaded with glucose, in a dose-dependent manner [99, 100]. The antihyperglycemic activities of this plant can be due to a number of factors such as decreased intestinal glucose absorption, increase in pancreatic secretions, glucose uptake, insulin secretion, and better glycemic control. Similarly, the antihyperglycemic activity of seeds and pericarps of *S. apetala* fruits were reported in STZ-induced diabetic mice [101]. The antihyperglycemic activity of this plant may be due to insulin mimetic activity, better glucose utilization, regeneration of islets of Langerhans in the pancreas, and enhanced transport of blood glucose to the peripheral tissue.

#### 2.2.13. Myrsinaceae

*A. corniculatum* belongs to the family Myrsinaceae in the mangrove communities and is commonly known from Indian mangroves. Several parts of the plant have been traditionally used for the treatment of inflammation, antioxidant, rheumatism, arthritis, free radical scavenging, and hepatoprotective activities [102]. The previous report revealed that ethanolic extract of *A. corniculatum* leaves regulates blood glucose level in alloxan-induced diabetic rats at a total of 100 mg/kg. Development in body weight in the diabetic-induced rat was observed along with a decrease in the activities of glucose-6-phosphatase, fructose-1,6-bisphosphatase and glycosylated hemoglobin along with the increased activity of liver hexokinase [37, 56].

#### 2.2.14. Meliaceae

Meliaceae is represented by two genera, namely, *Xylocarpus* and *Aglaia* in mangroves. The genus *Xylocarpus* is represented by three species, namely, *X. granatum, X. moluccensis,* and *X. rumphii* in India. Of these *X. granatum* and *X. moluccensis* are true mangrove species, whereas *X. rumphii* is a nonmangrove species [1]. In India, all three species are known to occur on the coast of ANI. Srivastava et al. reported that antidiabetic activities are exhibited by *X. granatum* and *X. moluccensis* [75]. Recently, Akter et al. revealed that methanolic extract showed antibacterial, antioxidant, and cytotoxicity potential with low concentration [103]. The ethyl acetate fraction of epicarp showed antidyslipidemic effects in diabetic-induced rats [104].

### 2.3. Diabetes Remedies from Traditional Knowledge on Mangrove Plants

Diabetes is one of the major public health concerns all over the world. Diabetes or hyperglycemia is considered to be one of the common public health hazards; optimal control of which is still not possible. Persistent hyperglycemia or uncontrolled diabetes has the potential to cause serious complications such as kidney disease, vision loss, cardiovascular disease, and lower-limb amputations which contributed towards morbidity and mortality in diabetes [105]. In India, traditional remedies have been used in the treatment of diabetes since the time of physicians Charaka and Sushruta. From the ethnobotanical recording, it is estimated that about 800 plants may possess antidiabetic potential [35]. Several plants have been used as a dietary adjuvant and in treating diseases even without any knowledge on their proper functions and constituents ref. This practice may be due to its fewer side effects compared to the synthetic hypoglycemic agents and because of their safety, effectiveness, and availability [6]. Ethnobotanical information reports a huge number of plants that may possess antidiabetic potential, of which *Momordica charantia*, *Pterocarpus marsupium*, and *Trigonella foenum-graecum* have been reported to be beneficial for the treatment of Type 2 diabetes. Throughout the world, terrestrial plants have been used in the treatments of diabetes. In contrast, very limited works have been carried out on the antidiabetic property of mangroves plants from India as well as globally.

Recently, ethnopharmacological records divulged the traditional usages of mangrove *A. corniculatum* (Linn.) Blanco distributed in coastal and estuarine areas of Southeast India [102]. Traditionally, more than 100 numbers of mangroves and mangrove-associated plant used for the treatment of diabetes have been reported, but only a very few numbers of plants are evaluated and reported scientifically [61]. The antidiabetic effect of leaves of mangrove plants *R. mucronata* and *C. decandra* had been documented and the gut perfusion studies on Long-Evans rats reported the mode of action of *R. mucronata* leaves' hypoglycemic conditions [32, 95]. Recently, the medicinal values of mangroves and associated plants persist to provide priceless therapeutic agents, both in modern medicines and in traditional systems [26].

## 3. Research Gaps in Indian Mangroves

In the past few decades, there is an increase in research works on mangrove plants in terms of conservation aspects especially from India. Mangrove species from several coasts all over the world have been studied for their medicinal values and for their bioactive potential to treat diseases like cancer, rheumatism, free radical scavenging, anti-inflammatory, antinociceptive, painful arthritis, inflammation, asthma, antioxidant, DM, and as hepatoprotective agents [102, 107].

**Figure 3:**
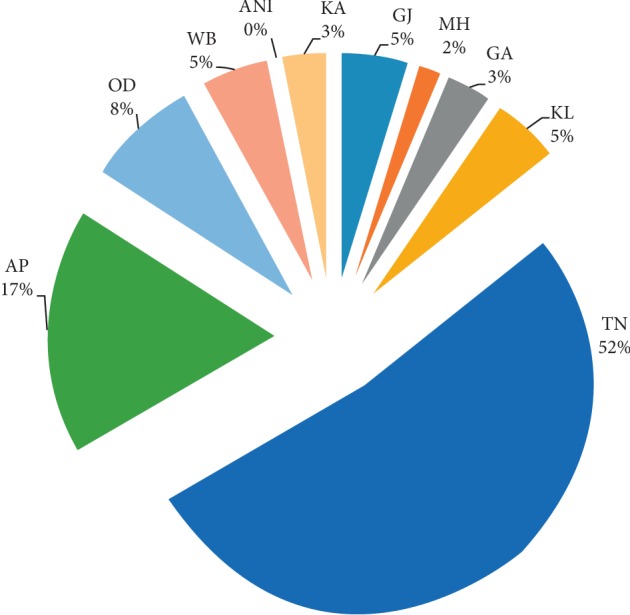


However, research on developing drug derivatives from mangroves by Indian scientific communities is very limited. Based on our data compilation on antidiabetic research, we suggest that research on mangroves is very much important as there are many potential medically significant compounds that have been reported from different regions, but very limited work has been carried out from the Indian coast, specifically on the phytochemical speciation. Further, there is a large gap in antidiabetic studies on ANI mangrove resources (Figure 3). Hence, antidiabetic studies in partnership with the indigenous communities of ANI based on their traditional knowledge are imperative.

## 4. Conclusions

In a nutshell, mangrove plants show high potential to address DM through their unique chemical structures. In recent years, most of the pharmaceutical industries are focusing mainly on the development of new drugs for DM on large-scale productions. But, there are promising potential from alternate sources like herbal medicines/traditional knowledge-based drugs which have multiple targets and potentially can be evolved as new drugs/complementary which needs serious attention. There is an important need to renew scientific research based on traditional knowledge of indigenous communities to be included in drug discovery programs. Medicinal valued plants are one of the chief components of our natural resource which was comprised of nearly 34 true mangrove species and 12 associated mangroves species from Indian coastal region. In order to enhance the anticipation, strategic selection of particular species and shortlisting of mangroves species is a necessary task for India. New research avenues on the traditional knowledge of medicinal plants may help to conserve time, money, and side effects (toxicity), which are three key major parameters that hurdle in any drug developmental program. Since TK is a community based knowledge, the historical laws of the indigenous community should be considered. Documented traditional knowledge on medicinal values of mangroves species might simplify issues associated with poor predictability and scientific research on such knowledge will create a new pathway for developing potential drugs against diabetes. Indian coastal communities have rich traditional knowledge on plant-based drug formulations that are protective and curative for many health ailments. However, there is a large research gap in antidiabetic studies on ANI mangrove resources with coastal communities based TK. In future, Indian tropical islands mangroves resources need to be studied in detail on antidiabetic compounds extraction for new drugs mining from unexplored pristine islands ecosystem.

## Figures and Tables

**Table 1:** Antidiabetics agents from mangroves plants.

Sl. No.	Chemical structure	Description	Reference
1.	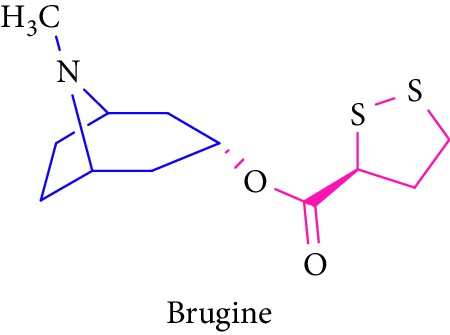	Source: *B. sexangula, B. cylindrica* (stem and bark) Mole. for: C_12_H_19_NO_2_S_2_ Mol. wt: 273.409 Biological activity: antidiabetic, anticancer	Katu and Takahashi [46]
			Richter et al. [47]
			Nebula et al. [48]
			Loder and Russell [50]

2.	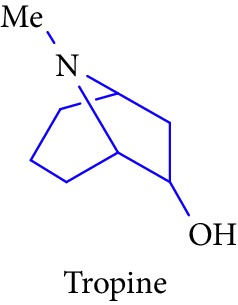	Source: *B. sexangular* (stem and bark) Mole. for: C_8_H_15_NO Mol. wt: 141.214 Biological activity: antidiabetic, anticancer	Loder and Russell [50]
			Brion et al. [51]

3.	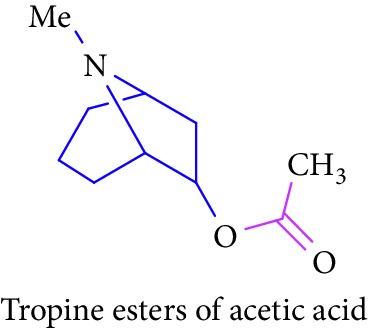	Source: *B. sexangular* (stem and bark) Mole. for: C_10_H_17_NO_2_ Mol. wt: 183.25 Biological activity: antidiabetic, anticancer, antiemetic, antispasmodics, mydriatics	Loder and Russell [50]
			Brion et al. [51]
			Gronkiewicz and Gadzikowska [52]

4.	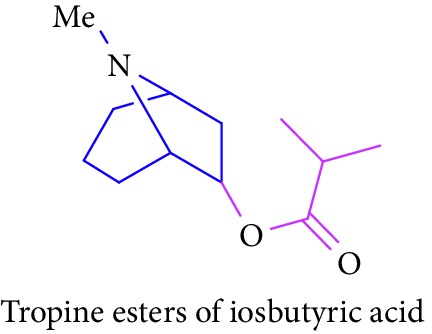	Source: *B. sexangular* (stem and bark) Mole. for: C_12_H_2_NO_2_ Mol. wt: 211.31 Biological activity: antidiabetic, anticancer, antiemetic, antispasmodics, mydriatics	Loder and Russell [50]
			Brion et al. [51]
			Gronkiewicz and Gadzikowska [52]

5.	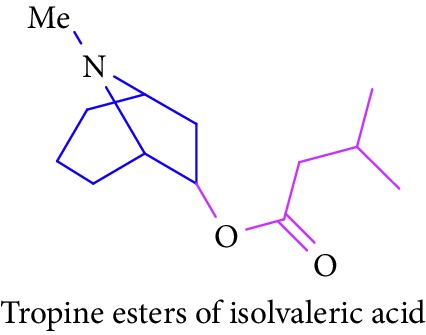	Source: *B. sexangular* (stem and bark) Mole. for: C_13_H_23_NO_2_ Mol. wt: 225.33 Biological activity: antidiabetic, anticancer, antiemetic, antispasmodics, mydriatics	Loder and Russell [50]
			Brion et al. [51]
			Gronkiewicz and Gadzikowska [52]

6.	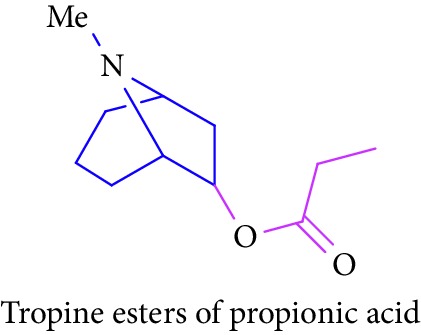	Source: *B. sexangular* (stem and bark) Mole. for: C_11_H_19_NO_2_ Mol. wt: 197.28 Biological activity: antidiabetic, anticancer, antiemetic, antispasmodics, mydriatics	Loder and Russell [50]
			Brion et al. [51]
			Gronkiewicz and Gadzikowska [52]

7.	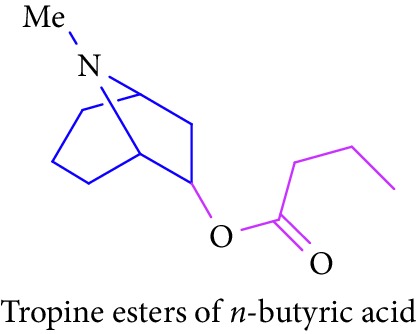	Source: *B. sexangular* (stem and bark) Mole. for: C_12_H_21_NO_2_ Mol. wt: 211.31 Biological activity: antidiabetic, anticancer, antiemetic, antispasmodics, mydriatics	Loder and Russell [50]
			Brion et al. [51]
			Gronkiewicz and Gadzikowska [52]

8.	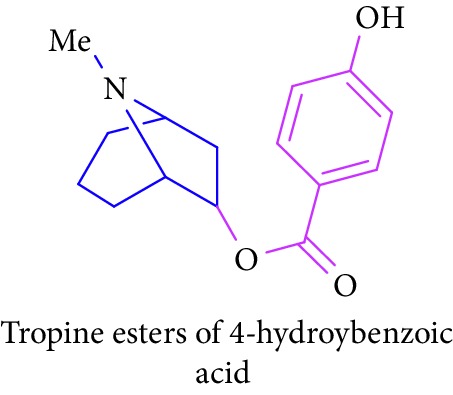	Source: *B. sexangular* (stem and bark) Mole. For: C_15_H_19_NO_3_ Mol. wt: 261.32 Biological activity: antidiabetic, anticancer, antiemetic, antispasmodics, mydriatics	Loder and Russell [50]
			Brion et al. [51]
			Gronkiewicz and Gadzikowska [52]

9.	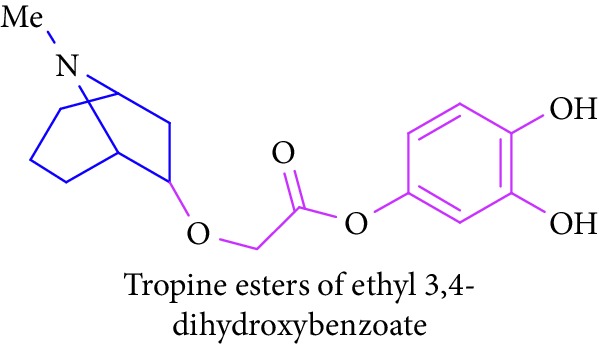	Source: *B. sexangular* (stem and bark) Mole. For: C_16_H_21_NO_5_ Mol. wt: 307.35 Biological activity: antidiabetic, anticancer, antiemetic, antispasmodics, mydriatics	Loder and Russell [50]
			Brion et al. [51]
			Gronkiewicz and Gadzikowska [52]

10.	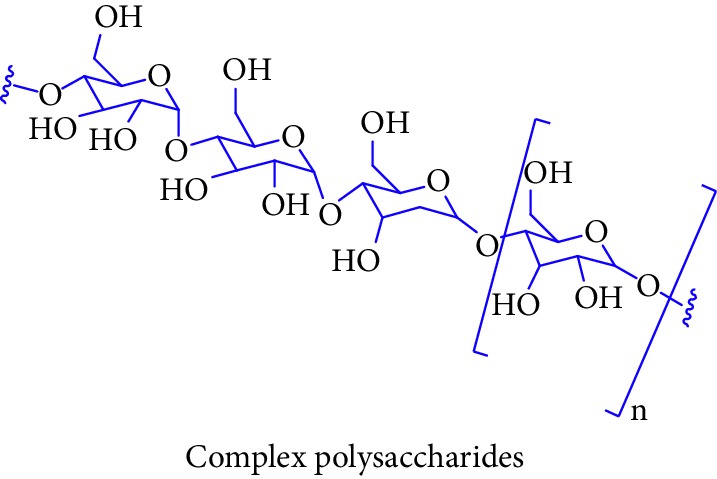	Source: *Sonneratia alba* Mole. for: C_*X*_ (H_2_O)_*Y*_ Mol. wt: 213–277 kDa Biological activity: antimicrobial, antiviral, antihyperglycemic agent, proliferation activity for fibroblasts	Liu et al. [53]
			Premanathan et al. [54]
			Morada et al. [55]
			Das et al. [56]

11.	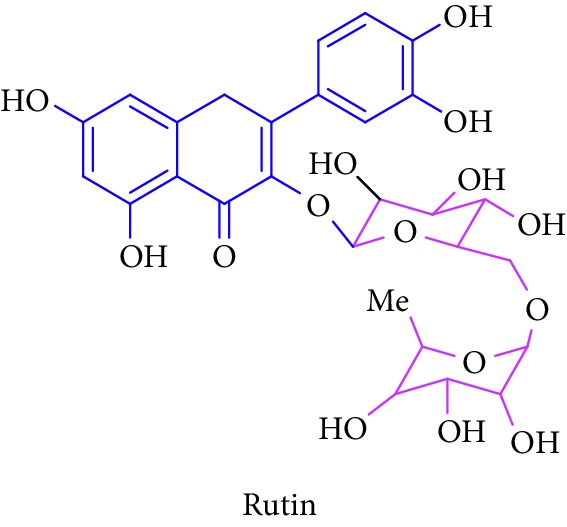	Source: *R. apiculata* and *A. ilicifolius, A. marina*, *X. granatum,* and *B. sexangula* Mole. for: C_27_H_30_O_16_ Mol. wt: 610.52 Biological activity: antidiabetic, antimicrobial, antiviral, antihyperglycemic, and proliferation	Kim et al. [49]
			Bisht et al. [57]
			Ganeshpurkar and Saluja [58]
			Kreft et al. [59]
			Harborne [60]
			Bandaranayake [61]
			Cheng et al. [62]
			Nebula et al. [48]

12.	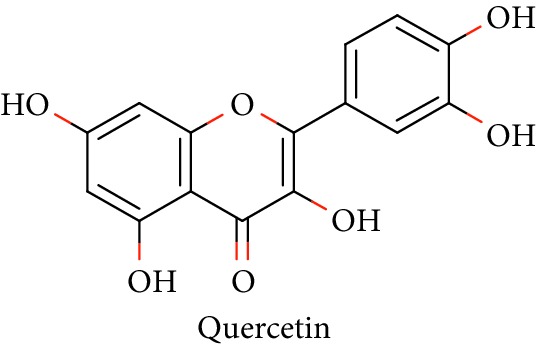	Source: *R. apiculata* and *A. ilicifolius, A. marina*, *X. granatum,* and *B. sexangula* Mole. for: C_15_H_10_O_7_ Mol. wt: 302.238 Biological activity: antidiabetic, antibacterial, antifungal, antimycobacterial, antimalarial, antiretroviral, antiviral	Kim et al. [49]
			Bisht et al. [57]
			Ganeshpurkar and Saluja [58]
			Kreft et al. [59]
			Harborne [60]
			Bandaranayake [61]
			Cheng et al. [62]
			Nebula et al. [48]

13.	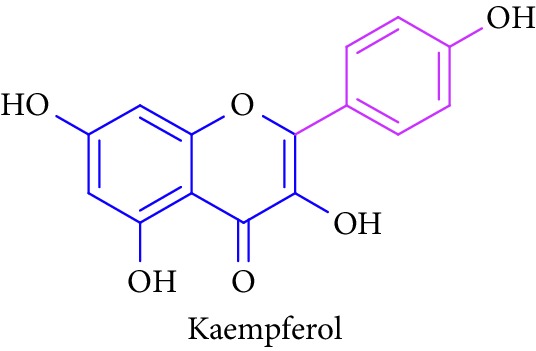	Source: *R. apiculata* and *A. ilicifolius, A. marina*, *X. granatum,* and *B. sexangula* Mole. for: C_15_H_10_O_6_ Mol. wt: 286.239 Biological activity: antidiabetic, anti-inflammatory, antihypertensive, vasodilator effects, antiobesity, antihypercholesterolemic, and antiatherosclerotic	Kim et al. [49]
			Bisht et al. [57]
			Ganeshpurkar [58]
			Kreft et al. [59]
			Harborne [60]
			Bandaranayake [61]
			Cheng et al. [62]
			Nebula et al. [48]

14.	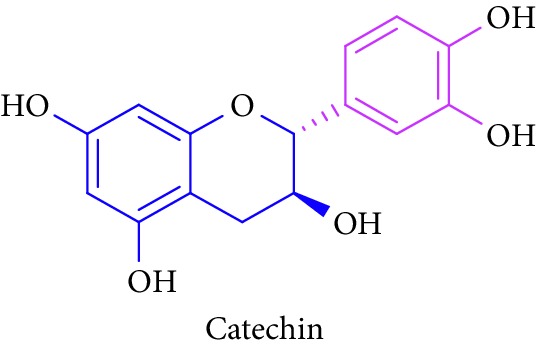	Source: *R. apiculata, A. ilicifolius, A. marina*, *X. granatum,* and *B. sexangula* Mole. for: C_15_H_14_O_6_ Mol. wt: 290.26 Biological activity: antidiabetic, antioxidant, anti-inflammatory, antimicrobial, anticancer, cardioprotective, neuroprotective, antiosteoporotic, estrogenic/antiestrogenic, anxiolytic, analgesic	Kim et al. [49]
			Bisht et al. [57]
			Ganeshpurkar and Saluja [58]
			Kreft et al. [59]
			Harborne [60], Bandaranayake [61]
			Cheng et al. [62]
			Nebula et al. [48]

15.	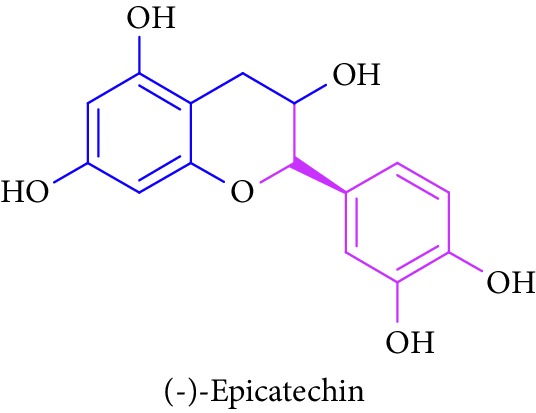	Source: *R. apiculata, A. ilicifolius, A. marina*, *X. granatum,* and *B. sexangula* Mole. for: C_15_H_14_O_6_ Mol. wt: 290.271 Biological activity: antidiabetic, antioxidant, anti-inflammatory, antimutagenic, cardiovascular disease	Kim et al. [49]
			Bisht et al. [57]
			Ganeshpurkar and Saluja [58]
			Kreft et al. [59]
			Harborne [60]
			Bandaranayake [61]
			Cheng et al. [62]
			Nebula et al. [48]

16.	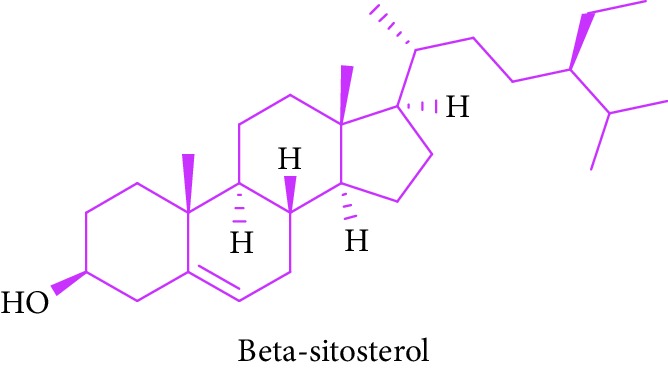	Source: *B. gymnorrhiza*, *B. sexangular* Mole. for: C_29_H_50_O Mol. wt: 414.71 Biological activity: antidiabetic, brain disorders, endothelial dysfunction, hypertension, neuroprotection	Nebula et al. [48]
			Vázquez et al. [63]
			Soodabeh et al. [64]

17.	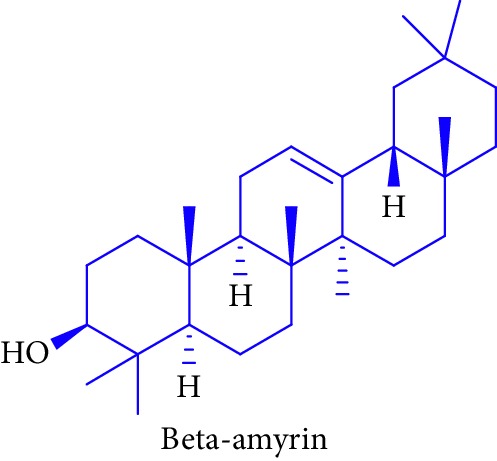	Source: *B. gymnorrhiza*, *B. sexangular* Mole. for: C_30_H_50_O Mol. wt: 426.729 Biological activity: antidiabetic, antioxidant, anti-inflammatory, and anticancer	Nebula et al. [48]
			Vázquez et al. [63]
			Soodabeh et al. [64]

18.	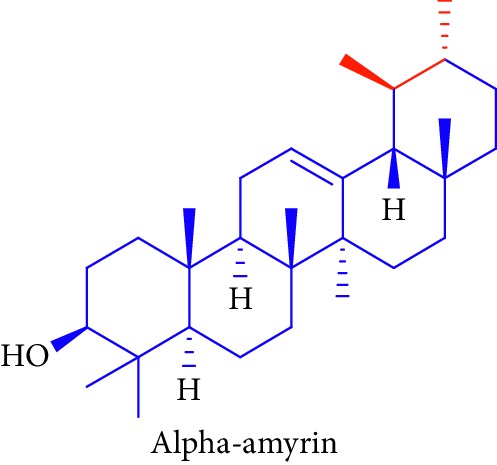	Source: *B. gymnorrhiza*, *B. sexangular* Mole. for: C_30_H_50_O Mol. wt: 426.729 Biological activity: antidiabetic, antinociceptive, anti-inflammatory	Nebula et al. [48]
			Vázquez et al. [63]
			Soodabeh et al. [64]

19.	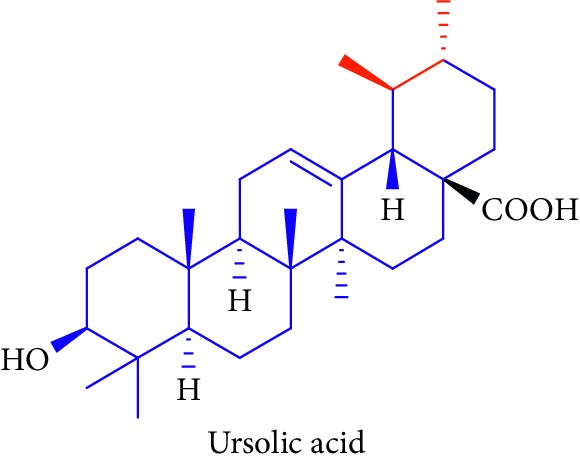	Source: *B. gymnorrhiza*, *B. sexangular* Mole. for: C_30_H_48_O_3_ Mol. wt: 456.7 Biological activity: antidiabetic, antinociceptive, and anti-inflammatory	Nebula et al. [48]
			Vázquez et al. [63]
			Soodabeh et al. [64]

20.	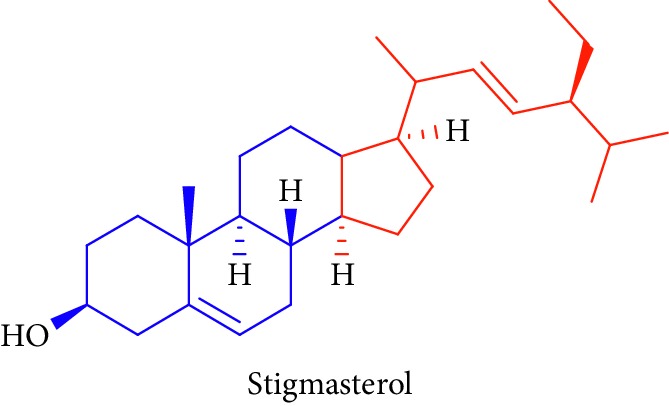	Source: *B. gymnorrhiza*, *B. sexangular* Mole. for: C_29_H_48_O Mol. wt: 412.69 Biological activity: antidiabetic, anticancer, anti-inflammatory, antioxidant, antihyperlipidemic, antihyperglycemic	Nebula et al. [48]
			Vázquez et al. [63]
			Soodabeh et al. [64]

21.	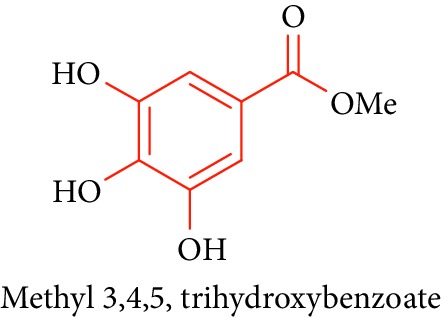	Source: *B. racemosa, Rhizophora* sp. Mole. for: C_8_H_8_O_5_ Mol. wt: 184.147 Biological activity: antidiabetic, anticancer, antidiabetes, anti-inflammation, antiosteoporosis, antipsoriasis, hepatoprotection, and hypolipidemic activity	Kabir et al. [65]
			Shahriar et al. [66]
			Saeki et al. [67]
			Li et al. [68]

22.	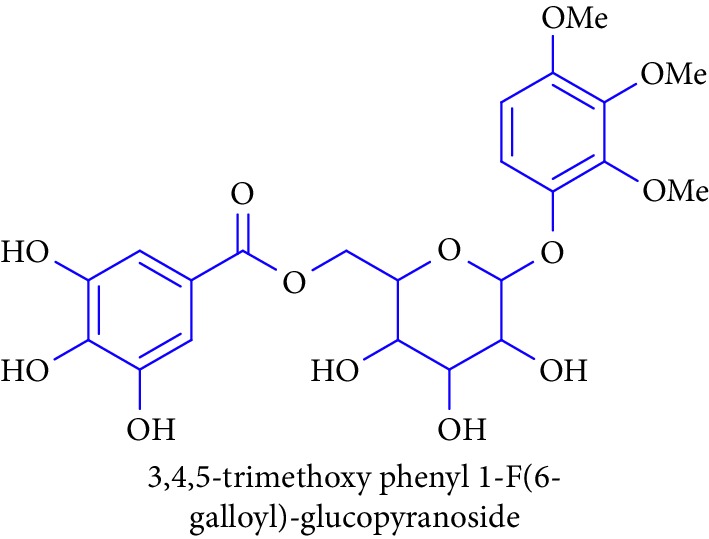	Source: *B. racemosa, Rhizophora* sp. Mole. for: C_22_H_26_O_13_ Mol. wt: 498.44 Biological activity: antidiabetic, anticancer, antidiabetes, anti-inflammation, antiosteoporosis, antipsoriasis, hepatoprotection, and hypolipidemic activity	Kabir et al. [65]
			Shahriar and Robin [66]
			Saeki et al. [67]
			Li et al. [68]

23.	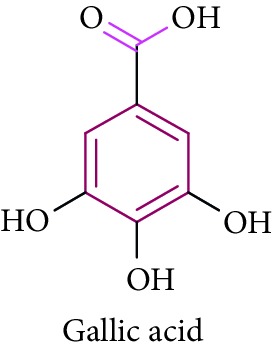	Source: *B. racemosa, Rhizophora* sp. Mole. for: C_7_H_6_O_5_ Mol. wt: 170.12 Biological activity: antidiabetic, antiherpetic, antioxidant	Kabir et al. [65]
			Shahriar and Robin [66]
			Saeki et al. [67]
			Li et al. [68]

24.	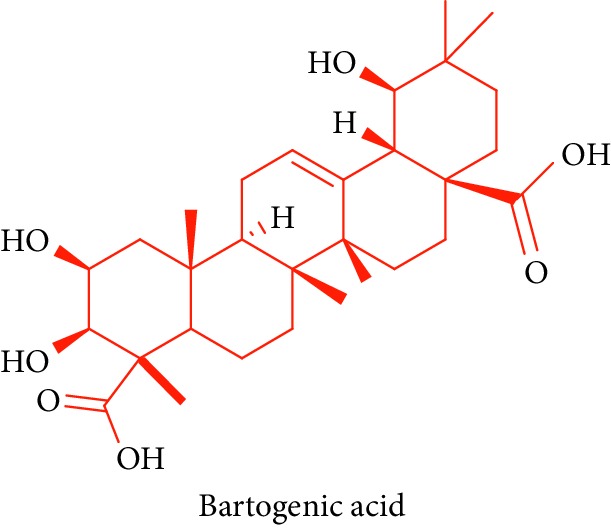	Source: *B. racemosa* (stem, bark, and fruits) Mole. for: C_30_H_46_O_7_ Mol. wt: 518.691 Biological activity: antidiabetic, antiosteoarthritic, antihypercholesterolemic, cytotoxicity, antitumor, hypoglycaemic, antimutagenic, antioxidant, anti-inflammatory, and CNS effects	Sun and Guo [69]
			Patil and Patil [70]
			Patil et al. [71]

25.	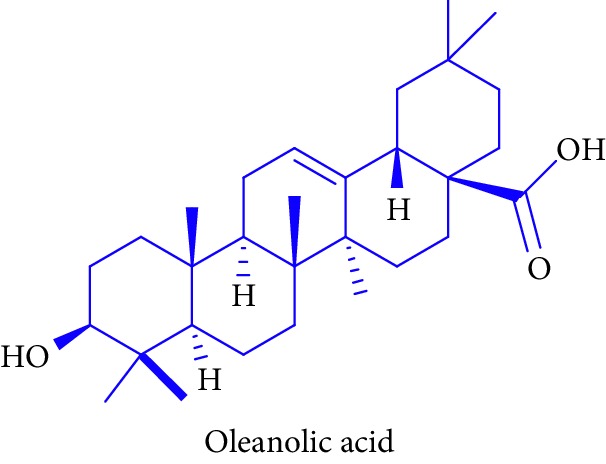	Source: *S. hydrophylacea* Mole. for: C_30_H_48_O_3_ Mol. wt: 456.711 Biological activity: antidiabetic, antiarthritic activity, antitumor, antinociceptive, antibacterial, and antifungal activities and anti-inflammatory drugs	Samarakoon et al. [72]
			Babalola et al. [73]
			Kurek et al. [74]

**Table 2:** Mangrove plants with phytochemical constituents and antidiabetic mechanism from different ecosystem (worldwide).

Sl.No.	Mangrove species	Phytochemical constituents	Antidiabetic mechanism	References
1.	*A. corniculatum*	Flavonoids, tannins, polyphenols Alkaloids, tannins, benzofurans Saponins	Utilization of glucose either by direct stimulation of glucose uptake or via the mediation of enhanced insulin secretion Presence of antidiabetic properties	Gurudeeban et al. [36]
				Ishibashi et al. [76]

2.	*Acrosathe annulata*	Amino acids inorganic salts	Presence of antidiabetic properties	Popp [77]

3.	*A. ilicifolius*	Flavonoids	Regeneration of *β*-cells of the pancreas Presence of flavonoids	Venkataiah et al. [78]
				Li et al. [68]

4.	*A. marina*	Saponins Yet to be analysed	Stimulation of *β*-cells to release more insulin antiglycation activity Presence of antidiabetic properties	Babuselvam et al. [118]
				Aljaghthmi et al. [79]

5.	*B. cylindrical*	Flavonoids, phenolic acids, sterols/triterpenoid, alkaloids, tannins, anthocyanins	Stimulation of *β*-cells to release more insulin	Das et al. [56]

6.	*Bruguiera* sp.	Alkaloids Flavonoids	Presence of antidiabetic properties	Cheng et al. [62]
				Li et al. [68]

7.	*B. racemosa*	Flavonoids, tannins, saponins Phenolic compounds	*α*-glucosidase and *α*-amylase inhibitory property Presence of antidiabetic properties	Gowri et al. [80]
				Kabir et al. [65]
				Li et al. [68]

8.	*B. gymnorrhiza*	Flavonoids, tannins, saponins, polyphenols, glycosides	Presence of antidiabetic properties	Nebula et al. [48]

9.	*B. rumphii*	Tannins, triterpenes	Presence of antidiabetic properties	Rollet [81]

10.	*B. parviflora*	Phenolic compounds	Presence of antidiabetic properties	Seshadri and Venkataramani [82]

11.	*B. sexangula*	Phenolic, steroids Alkaloids, tannins Saponins	Presence of antidiabetic properties	Hogg and Gillan [83]
				Nebula et al. [48]

12.	*C. decandra*	Flavonoids, tannins, saponins, polyphenols, glycosides	Presence of antidiabetic properties; stimulation *β*-cells to release more insulin The increased hexokinase activity	Seshadri and Trikha [84]
				Nabeel et al. [32]

13.	*C. tagal*	Flavonoids, tannins, saponins, polyphenols	The inhibition against PTPase enzyme activity	Tiwari et al. [85]
				Tamrakar et al. [86]
				Lawag et al. [87]

14.	*E. agallocha*	Flavonoids, tannins, saponins, polyphenols	Pancreatic secretion of insulin, uptake of glucose	Rahman et al. [88]
				Thirumurugan et al. [89]

15.	*K. candel*	Flavonoid, glycosides, triterpenoids, tannins, saponins, polyphenols	Presence of antidiabetic	Habeebulla and Velraj [90]

16.	*R. annamalayana*	Alkaloids, tannins steroids	Improved level of insulin secretion and its action	Ali et al. [91]
				Nabeel et al. [92]

17.	*R. apiculata*	Tannin, steroids, triterpenes, phenolic compounds	Improved level of insulin secretion and its action; insulin mimetic activity; cell protection	Lakshmi et al. [93]
				Sur et al. [55, 57, 58, 61, 75–77, 80–84, 90, 92, 94–113]
				Nabeel et al. [92]

18.	*R. mangle*	Tannins, triterpenes	Presence of antidiabetic properties	Willians [109]

19.	*R. mucronata*	Tannin, steroids, triterpenes, phenolic compounds	Improved level of insulin secretion and its action; insulin mimetic activity; *α*-glucosidase inhibitory	Nabeel et al. [92]
				Adhikari et al. [110]
				Aljaghthmi et al. [79]

20.	*R. racemosa*	Tannins, triterpenes	Presence of antidiabetic properties	Padmakumar and Ayyakkannu [111]

21.	*R. conjugate*	Anthocyanins, steroids Tannins, triterpenes	Presence of antidiabetic properties	Majumdar and Patra [112]

22.	*R. stylosa*	Inositols Steroids	Presence of antidiabetic properties	Ravi and Kathiresan [113]

23.	*N. fruticans*	Alkaloids glycosides tannins Sterols	Utilization of glucose	Reza et al. [114]

24.	*S. alba*	Tannins Phenolic Polysaccharides	Modifying glucose pathway Presence of antidiabetic properties	Morada et al. [55]
				Bandaranayake [61]

25.	*S. apetala*	Triterpenes steroids flavonoids alkaloids	Enhanced insulin-releasing activity; enhance transport of blood glucose to the peripheral tissue	Hossain et al. [101]
				Patra et al. [115]

26.	*S. brachiata*	Steroids Triterpenes	Presence of antidiabetic properties	Padmakumar et al. [116]

27.	*S. caseolaris*	Steroids glycosides	Intestinal *α*-glucosidase inhibitory activity; potentiation pancreatic secretion of insulin	Tiwari et al. [98]
				Hasan et al. [100]

28.	*S. ovata*	Steroids	Presence of antidiabetic properties	Bhosle et al. [117]

29.	*V. adenantha*	Yet to be analysed		Habeebulla and Velraj [90]

30.	*X. granatum*	Alkaloids steroids tannins triterpenes Alkaloids Flavonoids	Stimulation on *β*- cells; elevation in insulin sensitivity to glucose; protein tyrosine phosphatase activity Presence of antidiabetic properties	Srivastava et al. [75]
				Cheng et al. [62]
				Li et al. [68]

31.	*X. moluccensis*	Alkaloids steroids tannins triterpenes Proanthocyanidins	Insulin mimetic or insulin secretagogue activity insulin resistance reversal activity *α*-glucosidase inhibitory activity	Srivastava et al. [104]

## Abbreviations

AGE: Advanced glycation end-products
ANI: Andaman and Nicobar Islands
Alk-CF: Alkaline chloroform fraction
DOCK: Molecular docking
DCM-F: Dichloromethane fraction
DM: Diabetes mellitus
DPP: Dipeptidyl peptidase
ELISA: Enzyme-linked immunosorbent assay
GC-MS: Gas chromatography–mass spectrometry
HbA1c: Glycated hemoglobin
Hb: Hemoglobin
IUCN: International Union for Conservation of Nature
kg: kilogram
MCF-7: Michigan Cancer Foundation-7 breast cancer cell line
mg: milligram
NCI-H-292: Nonsmall lung cancer
NIDDM: Noninsulin-dependent diabetes mellitus
NIST: National Institute of Standards and Technology
PTPase: Protein tyrosine phosphatases
PAGE: Polyacrylamide gel electrophoresis
TK: Traditional knowledge
TM: Traditional medicines
SDS: Sodium dodecyl sulfate
STZ: Streptozotocin
WHO: World Health Organization
USA: United States of America
US: United States.
